# Don’t Call It Smart: Working From Home During the Pandemic Crisis

**DOI:** 10.3389/fpsyg.2021.741585

**Published:** 2021-09-30

**Authors:** Barbara Barbieri, Silvia Balia, Isabella Sulis, Ester Cois, Cristina Cabras, Sara Atzara, Silvia De Simone

**Affiliations:** ^1^Department of Political and Social Sciences, University of Cagliari, Cagliari, Italy; ^2^Department of Economics and Business, University of Cagliari, Cagliari, Italy; ^3^Department of Pedagogy, Psychology and Philosophy, University of Cagliari, Cagliari, Italy

**Keywords:** COVID-19, JD-R model, work from home, personal resources, quality of life

## Abstract

The recent COVID-19 pandemic and related social distancing measures have significantly changed worldwide employment conditions. In developed economies, institutions and organizations, both public and private, are called upon to reflect on new organizational models of work and human resource management, which - in fact - should offer workers sufficient flexibility in adapting their work schedules remotely to their personal (and family) needs. This study aims to explore, within a Job Demands-Resources framework, whether and to what extent job demands (workload and social isolation), organizational job resources (perceived organizational support), and personal resources (self-efficacy, vision about the future and commitment to organizational change) have affected workers’ quality of life during the pandemic, taking into account the potential mediating role of job satisfaction and perceived stress. Using data from a sample of 293 workers, we estimate measurement and structural models, according to the Item Response Theory and the Path analysis frameworks, which allow us to operationalize the latent traits and study the complex structure of relationships between the latent dimensions. We inserted in the model as control variables, the socio-economic and demographic characteristics of the respondents, with particular emphasis on gender differences and the presence and age of children. The study offers insights into the relationship between remote work and quality of life, and the need to rethink human resource management policies considering the opportunities and critical issues highlighted by working full-time remotely.

## Introduction

The COVID-19 has posed an unprecedented challenge to the global workforce. The lockdown experience and the prolonged confinement due to the persistence of the circulation of the virus, has determined important upheavals and transformations that have involved people and collective subjects at various levels. The pandemic has certainly acted as a powerful accelerator in the change of work and organizational processes and practices, pushing towards a rapid reconfiguration of the usual working objects, and the overcoming of traditional work-life boundaries. This sudden acceleration towards a much more intense use of remote work, although forms of smart working were being experimented in the Italian working context, has caught organizations and workers unprepared to manage this passage, considering the scarcity of alternative managerial models, guidelines, and policies recommending how to best move from the emergency response of the labor market to the diffusion of the virus, to a medium-long term solution of good integration of remote working into conventional work arrangements. Basically, what millions of Italian workers have witnessed and experienced in the last 16 months has been working from home or working remotely, without the necessary tools that configure a job that can be defined remotely or smart (Ripamonti et al., 2020). Furthermore, working from home has meant for many workers, especially in periods of isolation or partial confinement, to face a complete overlap between work and private life, and the acceptance of the loss of their role boundaries to cope with health emergency (Duffy, 2020). Work-related demands have invaded the family domain, at a time when the private domain has required immense effort in terms of childcare, housework, and family responsibilities (Vaziri et al., 2020). This situation has a particularly large impact on working parents and has produced a notable emotional impact on the general population, with important symptoms of anxiety, stress, and depression (Kang et al., 2020; Wang et al., 2020). To the authors’ knowledge, there are currently no studies in Italy that have explored the psychological implications of working from home, in terms of its effect on the perception of quality of life, although there are studies that have explored other factors related to remote work on the well-being of workers (Manuti et al., 2020; Molino et al., 2020; Ingusci et al., 2021; Ramaci et al., 2021; Valenti et al., 2021; Zammitti et al., 2021). The individual’s perception of quality of life can be defined, at least in part, as the cognitive appraisal of the distance between one’s standards, expectations, and goals, and the perceptions of the results achieved in the various domains of life (such as work, family, friendship), but also achieved in the past and achievable in the future (Rice et al., 1985). The quality of life is linked to interpersonal relationships on and outside of the job and can be considered crucial resources to managing stress in the workplace (Nappo, 2020). The stringent requests to change behaviors and lifestyles to contain the spread of the pandemic and the radical change in how people work (remotely and from home) have probably impacted people’s perception of quality of life. Using the JD-R theoretical framework (Demerouti et al., 2001; Schaufeli and Bakker, 2004; Bakker and Demerouti, 2007), the study aims to explore some specific job demands (i.e., workload, social isolation) and organizational and personal resources particularly significant in this moment (i.e., perceived organizational support, self-efficacy, vision about future, and commitment to organizational change) that may have influenced the quality of life during the lockdown and partial confinement phase, and to what extent some conditions (i.e., job satisfaction, perceived stress) have exerted a mediating effect.

## Literature Review and Hypotheses Development

### Demands and Resources in the Time of the Coronavirus

Literature offers different psychological models that clarify how work stress impacts the quality of life. For this study, we have chosen the Job Demands-Resources (JD-R) Model (Bakker and Demerouti, 2007), which classifies specific risk factors associated with job stress into two main categories: job demands and job resources. The Bakker and Demerouti (2007) job demands–resources model (JD-R model) is a transactional model that has been used to examine a variety of working environments or professions and assumes the simultaneous occurrence of job demands and job resources. Job demands are defined as “physical, psychological, social, or organizational aspects of the job that require sustained physical and/or psychological (cognitive and emotional) effort or skills” (Bakker and Demerouti, 2007, p. 312). Bakker and Demerouti (2007) further defined job resources as “physical, psychological, social, or organizational aspects of the job that are either functional in achieving work goals, reducing job demands and the associated physiological and psychological costs, or [in] stimulating personal growth, learning, and development” (p. 312). The JD-R model is an effective lens for examining the dynamic relationship between stress and resilience during the COVID-19 pandemic. This model has undergone revision (Demerouti et al., 2001; Bakker and Demerouti, 2007). Its revised version (Bakker and Demerouti, 2007), which is used in the current study, is improved over the previous version by considering internal resources called ‘personal resources’ (Xanthopoulou et al., 2007; Bakker and Demerouti, 2017; Taris et al., 2017). Personal resources may are conceptualized as strengths or characteristics that contribute to individuals’ optimal functioning (Youssef and Luthans, 2007). An advantage of the JD-R model (Bakker and Demerouti, 2007) is that it is responsive to demands in a variety of contexts and has the flexibility to incorporate variables that are unique or relevant to a specific context. Working from home during the COVID-19 pandemic certainly helped manage social distancing and consequently helped control the spread of the virus (Di Domenico et al., 2020; Kawashima et al., 2020). However, most organizations lacked a formal smart working policy and were not prepared for a general shift to remote working (Carnevale and Hatak, 2020; Rudolph et al., 2021). Working full time remotely was undoubtedly an unprecedented event for organizations and employees, who were forced, given the situation, to rethink and reorganize their work. As this is an unprecedented situation, there are no researches that have focused in the past on “mandatory,” full-time remote work. Furthermore, working from home full-time implies new and different demands and job resources, even the nature of existing demands and resources changes when work is brought home from the workplace for an ongoing period. Although a myriad of factors could potentially contribute to the quality of life of employees during the current pandemic, specific variables within three main categories were examined in the present study: job demands, organizational-job resources, and individual resources. Moreover, given the large body of research on remote working (e.g., Konradt et al., 2003; Grant et al., 2013), it can easily be assumed that workers necessarily placed to work remotely have faced psychological challenges and risks, due to different management of tasks, relationships, time dedicated to working and workspace.

### Job Demands Working at Home During a Pandemic: Workload and Social Isolation

Recent studies have shown that employees in remote working tend to work longer and harder (Kelliher and Anderson, 2010; Felstead and Henseke, 2017). In the pandemic scenario, remote working is no longer sensitive to employee preferences, flexible in time and space, but on the contrary, it is mandatory, and employees have no choice but to work full time from home. Wu and Chen (2020) for example have found that working from home not only increases employees’ workload, but also they constantly lose productivity due to stress and pressure. While the study by Kunze et al. (2020) highlighted that switching to work from home has tended to increase workloads, resulting in exhaustion. Furthermore, it can be hypothesized that people may be asked to work extra hours in the absence of commuting (Jamal et al., 2021) and that they may be involved in activities that depend on technological tools that are not always efficient or with which people can feel insecure, incapable, not able to use them (Ingusci et al., 2021). Finally, the implementation of remote work has led to an overlapping of work and family roles for a prolonged time and in the home environment, generating, in many cases, the need to manage a greater workload (Couch et al., 2021). Several studies of previous quarantine episodes have shown that psychological stress reactions may emerge from the experience of physical and social isolation (Brooks et al., 2020). Social isolation has often been put forward as the biggest disadvantage of remote working with more serious effects in the case of full-time remote working since it highly curtails opportunities for social interaction among employees (Golden et al., 2008; Morganson et al., 2010). Previous studies underlined that social isolation is generally associated with lower life satisfaction (Harasemiw et al., 2018; Zheng et al., 2020), higher levels of depression, and lower levels of psychological wellbeing (Cacioppo and Cacioppo, 2014; Coutin and Knapp, 2017; Harasemiw et al., 2018; Lee and Cagle, 2018; Usher et al., 2020). As already mentioned, working from home was necessary to ensure physical distancing in order to reduce the spread of COVID-19. However, this has intensified workers’ perceptions of being socially isolated. Several studies have highlighted how social isolation can negatively affect both mental and physical health and the overall quality of life of people (Cava et al., 2005; Berg-Weger and Morley, 2020; Brooks et al., 2020; Smith and Lim, 2020; Usher et al., 2020; Clair et al., 2021). The present study has considered workload (Brammer and Clark, 2020) and social isolation (Windeler et al., 2017) as the two relevant job demands in this working scenario conditioned by the pandemic situation. In light of the above, in this study, we hypothesized that:

H1. There exists a direct and positive relationship between job demands viz. workload (H1a), social isolation (H1b), and the employees’ perceived stress.

H2. There exists a direct and negative relationship between job demands viz. workload (H2a), social isolation (H2b), and the employees’ job satisfaction.

### Job Resource Working at Home During a Lockdown: Perceived Organizational Support, Self-Efficacy, Vision About the Future, Commitment to Change

Job resources refer to those physical, psychological, social, or organizational aspects of the job that are either/or: functional in achieving work goals; reduce job demands and the associated physiological and psychological costs; stimulate personal growth, learning, and development. Hence, resources are not only necessary to deal with job demands, but they also are important in their own right. As literature highlighted, job resources may be located at different levels: at the organization at large, at the interpersonal and social relations, at the organization of work, and at the level of the task (Bakker and Demerouti, 2007). In this exceptional situation, we imagined that the perception of feeling supported by one’s organization in managing new ways of working was an important resource capable of helping people to balance personal and professional life compromised by the pandemic situation. In fact, when employees receive resources, which they value high, it develops a positive perception for organizational support and they feel obligated towards the organization (Eisenberger et al., 2002; Rhoades and Eisenberger, 2002). This is the reason why in this study we hypothesized that organizational support could be a resource that might influence the quality of life. JD-R traditionally focuses on characteristics of the job as demands and resources. However, recent research moves toward considering also the role of the individual as a “job crafter” (Bakker et al., 2012; Hakanen et al., 2017; Petrou et al., 2017), because individuals bring personal resources to bear on the work situation (Bakker et al., 2012; Xanthopoulou et al., 2012; Huang et al., 2016; Grover et al., 2017). Personal resources are aspects of the self that are generally linked to resilience and refer to individuals’ sense of their ability to control and impact their environment successfully (Xanthopoulou et al., 2007, pp. 123–124). Thus, in light of above, personal resources can be important determinants in facilitating a process of adaptation to the new working conditions (Hobfoll, 1989; Judge and Cable, 1997) and the acceptance of a so radical change in working practices. Personal resources can form stronger positive evaluations about themselves (Xanthopoulou et al., 2007) and this could make it easier to cope with adverse or difficult working conditions (such as the one we are experiencing). In other words, personal resources can determine how people perceive the work circumstances they are experiencing and react positively to them (Judge and Cable, 1997; Judge et al., 2000). If we apply this reciprocity perspective to the JD-R model, we can expect employees who perceive themselves to be self-effective (Bandura, 2010) and who can see beyond what is happening, having a more future-oriented time perspective (Ginevra et al., 2016), to focus more on working resources than on job demands and, as a result, they will experience lower levels of stress and higher levels of job satisfaction. Individuals with high self-efficacy select challenging tasks and higher goals, invest more effort, recover more quickly and persist longer (Bandura, 1997). Thus, self-efficacy is a crucial personal resource in coping with the challenges and demands in difficult and changing situations. Also, vision about the future can be considered as a personal resource because it has to do with the individual ability to code and distinguish the current emergency condition from the presumable and desirable restoration of a state of new stability towards which to strive, and to be taken as an objective in the medium term, often referred to as the “new normal” in public discourse. This would allow them, despite the difficulties, to maintain a good quality of personal life. Furthermore, in light of the timely request to switch from one working model (traditional) to another (remote), we felt that the commitment to change could be an important organizational resource in this situation. In fact, according to Meyer and Herscovitch (2001), commitment to change can be considered as a mindset that binds an individual to a course of action deemed necessary for the successful implementation of a change initiative. Therefore, in this study, we hypothesized that:

H3. There exists a direct and negative relationship between organizational and individual job resources viz. perceived organizational support (H3a), self-efficacy (H3b), vision about the future (H3c), commitment to change (H3d), and the employees’ perceived stress.

H4. There exists a direct and positive relationship between organizational and individual job resources viz. perceived organizational support (H4a), self-efficacy (H4b), vision about the future (H4c), commitment to change (H4d), and the employees’ job satisfaction.

H5. There exists a direct and positive relationship between individual job resources viz. self-efficacy (H5a), vision about the future (H5b), commitment to change (H5c), and the employees’ perception of quality of life.

### Perceived Stress and Job Satisfaction as Mediators Between Demands and Job Resources on the Quality of Life

The overlap between the antecedents of work stress and job satisfaction suggests that their mapping within the JD-R model should provide a means of benchmarking to identify the main influences on both phenomena simultaneously (McVicar, 2016). In the work context, perceived stress refers to the feeling of not being able to cope with work demands (Hobfoll, 1989; Lee and Ashforth, 1996). We can define stress as the depletion of the emotional and mental energy needed to do the job (Moore, 2000). Although job demands are not essentially negative, they can lead to stress due to the high efforts required to meet them (Sardeshmukh et al., 2012) and employees can feel stressed when perceived resources are inadequate to meet job demands (Wright and Cropanzano, 1998). While job satisfaction is an affective (emotional) response by an individual concerning his/her job that results from a comparison of actual outcomes with those that are expected, wanted, and needed (Griffin et al., 2010). Job satisfaction refers to pleasurable psychological experiences, which can lessen or eliminate some of the negative job demands. Those with high satisfaction may look forward to work and may be less troubled by strains from the job. Under the JD-R model, considering the pandemic scenario and the radical change in work, due to forced full-time work from home, these constructs should mediate the relationship between job demands and resources and people’s perceived quality of life. In light of above, we hypothesized that:

H6. Perceived stress mediates the relationship between job demands viz. workload (H6a), social isolation (H6b), and the employees’ perception of quality of life.

H7. Perceived stress mediates the relationship between organizational and individual job resources viz. perceived organizational support (H7a), self-efficacy (H7b), vision about the future (H7c), commitment to change (H7d), and the employees’ perception of quality of life.

H8. Job satisfaction mediates the relationship between job demands viz. workload (H8a), social isolation (H8b), and the employees’ perception of quality of life.

H9. Job satisfaction mediates the relationship between organizational and individual job resources viz. perceived organizational support (H9a), self-efficacy (H9b), vision about the future (H9c), commitment to change (H9d), and the employees’ perception of quality of life.

## Method

### Survey Data and Indicators

To test the hypotheses described we use data on Italian workers who, after receiving an invitation via social media (facebook), volunteered have been participate to an online survey by self-completing a questionnaire administered in July 2020. The anonymity of subjects was guaranteed according to the General Data Protection Regulation and the Helsinki Declaration (World Medical Association, 2013). Before completion of the questionnaire, individuals provided their informed consent. Data were computed in an aggregated manner without any possibility to identify the personal information of subjects. The anonymized questionnaire, which could be filled in about 20 min, contained a module investigating socio-economic and demographic characteristics and a module with questions focused on gathering information on job demands and resources, stress, job satisfaction and quality of life. Only workers who were forced to work from home during the Covid19 pandemic “great lockdown” were eligible to participate to the survey. A total of 293 Italian individuals aged 45.1 years old (s.d. 7.8), of which 73.7% are women, participated to the survey. Summary statistics are reported in Table 1. The level of education among participants is very high (79.2% of them has a bachelor/master or a post graduate degree); about 56.7% of them work in the public sector. Approximately half of the respondents (51%) has no children, while about 17.4% of them has children in pre-scholar age (under 6 years old); 22.9% has children in scholar age (6–18), and 8.5% has older children.

**TABLE 1:** Scale information (*n* = 293).

**Type of variable**	**Indicator role in Path Analysis**	**Scale**	**# item**	**# reversed items**	**# categories**	**Cronbach’s alpha**
Endogenous	Outcome	Quality of life	8	0	5	0.85
Endogenus	Mediator	Job satisfaction	4	1	7	0.89
Endogenous	Mediator	Stress	10	5	5	0.83
Exogenous	Job demand	Workload	10	1	5	0.89
Exogenous	Job demand	Social isolation	10	0	7	0.94
Exogenous	Job resource	Organizational support	8	4	5	0.9
Exogenous	Job resource	Vision about the future	19	4	5	0.95
Exogenous	Job resource	Self efficacy	10	0	5	0.9
Exogenous	Job resource	Commitment to change	18	11	5	0.87

### Measures

The following validated scales have been adopted in order to operationalize job demands and job-organizational and individual resources components and to assess the hypotheses advanced.

To evaluate *workload* construct we used 10 items extracted from three different tools measuring job stressor and strain. Specifically, Interpersonal Conflict at Work Scale (ICAWS) (Spector and Jex, 1998) that consists of 4 items rated on a 5-point Likert scale was employed. In addition, were used 5 items rated on a 5-point Likert scale from the Quantitative Workload Inventory (QWI) developed by Spector and Jex (1998). Finally, to measure time availability, we utilized one item from the Organizational Constraints Scale (OCS; Spector and Jex, 1998) that assesses the constraints areas discussed in Peters and O’Connor (1980).

*Workplace Isolation Inventory*, developed by Marshall et al. (2007), was used to measure two sub-dimensions, physical and informational isolation. It consists of 10 items selected and adapted from the original Workplace Isolation Inventory. Participants answered on a 7-point agreement scale (from 1 = strongly disagree to 7 = strongly agree). The physical isolation scale includes items such as “I am isolated from others at work,” instead, informational isolation scale is based on items such as “I often miss the opportunity to meet key people whom I work with.”

To measure *perceived organizational support*, we utilized the format for the 8-item Survey of Perceived Organizational Support Scale (SPOS) developed by Eisenberger et al. (1986). The scale includes 8 items rated on a 7-point agreement scale (from 0 = strongly disagree to 6 = strongly agree). Examples of items are “The organization really cares about my well-being,” or “The organization takes pride in my accomplishments at work.”

*Commitment to Organizational Change Scale*, (Herscovitch and Meyer, 2002), was used to assess the commitment to organizational change. The instrument consists of 18 items rated on a 5-point agreement scale (from 1 = strongly disagree to 5 = strongly agree) that provides a score of affective commitment, continuance commitment and normative commitment. Participants answered on items such as “I believe in the value of this change,” or “I feel a sense of duty to work toward this change.”

The Italian version of the *General Self-Efficacy Scale* (GSE) was used to assess self-efficacy construct. The scale, developed by Sibilia et al. (2019), includes 10 items rated on a 5-point agreement scale (from 1 = strongly disagree to 5 = strongly agree). A typical item is: “Thanks to my resourcefulness I can handle unforeseen situations.”

To measure the vision about the future we used the *Vision About the Future Scale* (VAF) developed by Ginevra et al. (2016). Participant answered on 19 items, rated on a 5-point Likert scale (from 1 = it does not describe me at all to 5 = it describes me very well), which assess hope, optimism and pessimism.

As mediator variables, we considered job satisfaction and perceived stress.

Job satisfaction was measured using the Brief Overall Job Satisfaction measure II developed by Judge et al. (1998). The scale is composed by 5 items rated on a 7-point agreement scale (from 1 = strongly disagree to 7 = strongly agree). The perception of satisfaction concerning the current job of the respondents was assessed using items such as “On most days I am enthusiastic about my work,” or “I consider my job rather unpleasant.”

Perceived stress was measured using the Italian 10-item version of the Perceived Stress Scale (IPSS-10) developed by Mondo et al. (2019). The scale measures thoughts and feelings related to stressful events through 10 items rated on a 5-point Likert Scale (from 0 = never to 4 = very often). Example of items are “in the last month, how often have you been/felt nervous and stressed?” or “in the last month, how often have you been/felt you were on top of things?”.

In this study, we explore quality of life of working individuals, using the definition proposed by the World Health Organization (WHO), which describes it “as an individual’s perception of their position in life in the context of culture and of the value systems in which he lives and in relation to his goals, expectations, standards and concerns.” This definition corresponds perfectly to the multidimensional concept we aim to measure. In particular, in this study we use the 8-point version of the WHO Quality of Life Index (also referred to as EUROHIS-QOL 8 index) that collects information on eight fundamental areas, which are particularly relevant in this study because they inspect domains such as general quality of life, general health status, energy for everyday life, ability to perform daily activities, self-esteem, personal relationships, financial hardship, and living conditions, as shown in Table 2 (for additional details, see Nosikov and Gudex, 2003). Each item has a 5-point response format on a Likert-scale to rate the respondents’ level of satisfaction in each domain. The EUROHIS-QOL 8 index is derived from the WHOQOL-BREF (26 items), which, in turn, is a shorter version of the original instrument, the WHOQOL-100, which is suitable for clinical and general population. It was proposed by the EUROHIS project with the goal of creating a common instrument to be used for quality of life comparisons between national cultures and within countries, overcoming the drawbacks of scales that were based on a narrow definition of “well-being” and that were not able to mix together both health and non-health determinants of quality of life. Rasch analysis, confirmatory and exploratory factor analyses were used to derive items that showed the best overall fit for a single factor (see Power, 2003 for further details). The psychometric properties of the EUROHIS-QOL 8-item index in terms of its reliability, convergent and discriminant validity, and in terms of its cross-cultural performance have been proved also by Schmidt et al. (2006) and by Schiavolin et al. (2015) in a study on adult Italian patients. The final index used to capture quality of life in our study is obtained using the posterior predictions of the person location parameters of the EUROHIS-QOL 8-item scale. Higher scores indicate better quality of life.

**TABLE 2:** EUROHIS-QOL 8-items in a sample of Italian workers interviewed during the Covid19 pandemic great lockdown (*n* = 293).

**Items**	**Mean**	**St. dev.**	**Item test correlation**	**Cronbach’s alpha (-j)**
How would you rate your quality of life	3.874	(0.832)	0.762	0.819
How satisfied are you with your health	3.819	(0.890)	0.651	0.836
Do you have enough energy for everyday life	3.935	(0.827)	0.790	0.814
How satisfied are you with your ability to perform your daily activities	3.659	(0.996)	0.547	0.851
How satisfied are you with yourself	3.874	(0.841)	0.745	0.822
How satisfied are you with your personal relationships	3.788	(0.838)	0.774	0.817
Have you enough money to meet your needs	3.778	(0.984)	0.661	0.835
How satisfied are you with the conditions of your living place	3.911	(0.986)	0.634	0.839

For all scales used in the analysis, negative items with respect to the direction of the name of the scale have been reversed, so that higher values of the operationalized variables signal a higher position of the respondents on the latent trait the scale intends to measure (e.g., higher values on the Perceived stress scale signal a worse position of respondents with respect to stress). Informations on the scale characteristics and on their degree of reliability are reported in Table 3. As shown by the high values of the alpha Cronbach coefficient, which varies between 0.83 to 0.95, all scales show a satisfactory level of reliability.

**TABLE 3:** Summary statistics (*n* = 293).

**Variable**	**Mean**	**Std. Dev.**	**Min**	**Max**
Quality of life	0.000	1.987	−6.950	4.553
Job satisfaction	–0.085	3.879	−11.498	6.912
Perceived stress	0.000	1.154	−3.019	3.156
Social isolation	0.000	1.980	−4.737	4.689
Workload	0.000	0.995	−2.681	2.849
Self-efficacy	–0.001	1.754	−4.287	3.355
Vision about future	–0.001	2.210	−6.560	5.566
Commitment to change	0.000	1.279	−4.132	3.125
Perceived organizational support	0.000	2.037	−5.308	4.998
Female	0.737	0.441	0	1
Age	45.150	7.935	25	65
Age squared (/100)	21.013	7.267	6.25	42.25
Married	0.727	0.446	0	1
Children (0–6)	0.174	0.380	0	1
Children (6–18)	0.229	0.421	0	1
Children (18 and older)	0.085	0.280	0	1
University degree	0.792	0.407	0	1
Employed in public sector	0.567	0.496	0	1

### Empirical Approach

**FIGURE 1:**
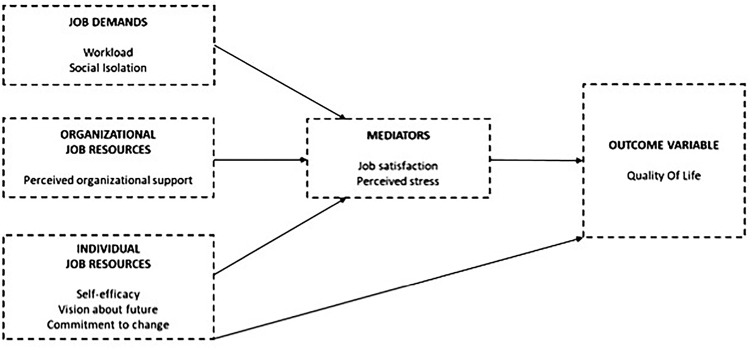
Hypothesized conceptual framework. A hybrid model for Quality of Life in a Latent Path Analysis framework.

In this study, we investigate the relationship between a specific set of job demands and resources on (health and non-health related) quality of life of workers at the time of the “great lockdown” imposed in Italy during the Covid-19 pandemic in 2020. Job demands and resources might influence the quality of life either directly or indirectly through their effect on perceived stress and job satisfaction, which, in turn, affect the quality of life. In addition, the disease itself and the non-pharmacological measures adopted to contain the virus might have influenced individuals’ perception of their roles in society, in the family, and in the workplace. We propose a two-step analysis that consists of a measurement approach for the operationalization of the latent traits and a path analysis to test the hypotheses described in Figure 1. The selection of this two- steps procedure was imposed by the low sample size that did not allow us to proceed with a Latent Path Regression Analysis. In Step 1, we use the Item Response Theory (IRT) to operationalize the latent variables for quality of life, a set of job demands and resources, perceived stress, and job satisfaction for which we have collected information using an *ad hoc* survey. IRT produces estimates of respondents’ location on the latent traits by considering the different characteristics of the categorical items composing each scale. IRT modeling approach (Baker and Kim, 2004; de Ayala, 2009) is one of the most complete measurement method for the development, refinement, and validation of scales when information is gathered by multi-item binary or categorical scales.

For ordered categorical items the Graded Response Model (GRM) (Samejima, 1969) is widely adopted since it is the natural extension of the most flexible model for binary responses, known as the two-parameter logistic model, 2PL, (Birnbaum, 1968). In its general formulation, GRM specifies the probability that respondent *i* (i = 1,…, n) provides a responses at least equal to *k* (k = 1,..,L) to an item *j* (j = 1,..,M) as follows:


$$P\left(Y_{i\,j}\geq k|\theta_{i},\tau_{j\,k},\alpha_{j}\right)=\frac{e^{\alpha_{j}\,\left(\theta_{i}-\tau_{j\,k}\right)}}{1+e^{\alpha_{j}\,\left(\theta_{i}-\tau_{j\,k}\right)}}$$

for k = 1,…, L–1, where τ_*jk*_ is the item-category (threshold) parameter of item *j* category *k*, α_*j*_ is the discrimination parameter and θ_*i*_ is the person parameter which is shared by responses to each scale provided by the same individual. The greater θ_*i*_ the higher the probability that respondents cross category *k* of item *j*, whereas the greater τ_*jk*_, the higher is the minimum required level of latent trait required to respondents in order to cross it. When θ_*i*_ = τ_*jk*_ this probability is equal 0.5. The model has been estimated using the routine SEM for Generalized Linear Mixed Models in Stata, which treats person parameters as random intercepts which follow a normal distribution (θ_*i*_∼*N*(0,σ^2^)) and τ_*jk*_ and α_*j*_ as fixed effects. The model has been estimated using Maximum Likelihood with adaptive Gauss–Hermite quadrature. A different parametrization of the equation 1 is used in the routine SEM in Stata which is implemented for structural equation models (see Acock, 2013; StataCorp, 2021). The estimate of individual position on the latent trait θ_*i*_ related to each single scale is obtained at posterior using empirical Bayes means predictions of the latent traits.

In Step 2, we estimate the structural part of the model using a Path analysis, which allows us to uncover the pathways from the JD-R variable to quality of life, disentangling the effects that run through the mediators, and explicitly account for respondents’ heterogeneity in socio-demographic characteristics (Acock, 2013; Finch and French, 2015). Our approach models the relationships between exogenous and endogenous variables by specifying a series of sequential regression equations, where mediators are dependent variables in auxiliary equations and predictors in the main equation for quality of life. The advantage of using this approach is that the effects of the exogenous variables on the outcome variables can be estimated considering both direct and indirect effects, where the latter depend on specific mediating variables (the so-called “partially mediated model”). The sign and size of the “path coefficients” are used to assess the strength of the estimated relationships and can be better interpreted as standardized slopes through the use of a correlation coefficient *r* (which ranges in interval −1; 1) when the attention is focused on the strength of the linear relationship between exogenous and endogenous variables. In this work, we estimate a “hybrid” specification of the path model, that shares the features of “a fully mediated model,” where the exogenous variable affects the endogenous outcome variable only through the mediators, and a “partially mediated model” as described above. Model fit is assessed using the Root Mean Squared Error Approximation (RMSEA) statistics, a goodness of fit measure which compares the observed (*satured model*) and the estimated (S hypothesized model) variance-covariance matrix in terms of deviance by accounting for the effective sample size and introducing a penalization factor which advantages more parsimonious models (Kline, 2015). Values of the RMSEA ≤ 0.05 highlight good model fit and thus support the advanced hypotheses, whereas a general convergence in the literature can be found in judging poor values of RMSEA greater than 0.08, and unacceptable models with RMSEA greater than 0.10.

## Results

The analysis has been carried out on the whole sample (*n* = 293) and, for comparative purposes, on the subsample of women (*n* = 216). Both samples support the model described in Figure 1. Results are reported in Supplementary Tables 1, 2.

The RMSEA indexes show a good level of model fit (0.05 and 0.02 for the subset of women and the whole sample, respectively). The r-squared coefficient of determinations (Bentler and Raykov, 2000) show, for both samples, that the set of predictors in the three equations explain about 57–58% of the share of variability in quality of life, about 55–59% of the variability in stress and 37–41% of the variability in job satisfaction. The overall model coefficient of determinations signals, that about 74–76% of the variability in quality of the life, is explained by the exogenous and mediating variables. All coefficients of determinations are larger for the women subgroup. The sign and the size of the slope coefficients support the hypotheses advanced. In our “hybrid” model, job satisfaction and perceived stress have a partially mediating effect when the block of predictors considered in the analysis are socio-demographic and individual job-resource covariates (supporting hypotheses H6: H9), where the latter factors have a total effect on quality of life that can be disentangled into a direct and indirect effect (supporting hypothesis H5). Furthermore, job satisfaction and perceived stress fully mediate the effects of job-demands and perceived organizational support on quality of life (supporting hypotheses H6 and H7), meaning that total and indirect effects coincide. Job-resources such as vision about the future, self-efficacy, and commitment to change have a positive effect on our measure of quality of life (supporting hypothesis H5). Specifically, self-efficacy and commitment to change have significant indirect effects on quality of life (H7b, H7d). Both are negatively associated with similar strength with perceived stress (*r* = −0.18; −0.16 in the whole sample and *r* = −0.14; −0.15 in the women subsample), whereas self-efficacy positively influences job-satisfaction (*r* = 0.15 and 0.20 in the two samples). Only commitment to organizational change, and vision about the future have a significant total effect on quality of life outcome endogenous variables. Vision about the future is the predictor with the strongest association with quality of life (*r* = 0.56). The indicators of job demand and organizational support used in the analysis influence quality of life only through the mediators (supporting hypotheses H1, H2, H3a and H4a combined with H7a and H9a). Isolation and total workload increase perceived stress (supporting hypothesis H3). Workload exerts a certain effect only through perceived stress (*r* = 0.17 in the whole sample and *r* = 0.19 in the women subsample), while organizational support contributes to a better quality of life only by means of its influence on job satisfaction (*r* = 0.32 in the whole sample and *r* = 0.36 in the women subsample). Overall, findings suggest that an increase in job demands – which is very likely to have occurred during the lockdown – has a negative impact on quality of life. As expected, the two mediator variables have a relevant and opposite influence on quality of life, with a stronger negative effect of stress (*r* = −0.39 in the whole sample and *r* = −0.40 in the women subsample) with respect to the positive impact of job satisfaction (*r* = 0.14 in the whole sample and *r* = 0.19 in the women subsample) (supporting hypotheses H5:H9). As shown by the women subsample estimation, results seem to be mainly driven by female workers. In general, socio-demographic characteristics mostly influence quality of life through their effects on stress and job satisfaction. Education, however, shows a direct beneficial effect: individuals with a university degree report a better quality of life. On average, having children younger than 18 years old has a positive effect on perceived stress (*r* = 0.10; 0.11 in the whole sample and 0.13 in the women subsample). For women the presence of children in pre-scholar age (0–6) also significantly reduces reported job satisfaction (*r* = −0.11).

## Discussion

The aim of this study was to explore, within a Job Demands-Resources framework (Bakker and Demerouti, 2007), whether and to what extent job demands (workload and social isolation), organizational job resources (perceived organizational support) and individual job resources (self-efficacy, vision about the future, and commitment to organizational change) have affected workers’ quality of life during the pandemic, taking into account the potential mediating role of job satisfaction and perceived stress. It also enhances our understanding of the effects of job resources and job demands on the individuals’ wellbeing that worked from home during the pandemic. First of all, the results provide evidence on how logistical and organizational change has passed on the perceived quality of life, in terms of satisfaction with one’s performative capacity and ability to resist and manage stress, precisely because the pandemic state of exception has redefined the overall workload, in the space-time overlap of domestic and professional tasks. As expected, job resources and demands are largely associated with stress and job satisfaction (supporting hypotheses H2-H4), and these in turn, mediate the effect of job demands and resource on quality of life (supporting hypotheses H8-H9). Overall, our findings suggest the importance that promoting job satisfaction and preventing work stress may have in fostering wellbeing and promoting quality of life, especially during a pandemic. The level of work stress, and its consequences, can be reduced and prevented by identifying its main sources, with a positive effect on both individual and organizational wellbeing. Managers and job analysts should identify which situations are most likely to trigger stress, identify the main sources of stress, during pandemic and in remote work, and plan ad hoc actions. Job satisfaction is an important positive dimension of wellbeing at work (Rothmann, 2008). Understanding the predictors of job satisfaction contribute to the improvement of wellbeing in times of pandemics. To improve job satisfaction, HRM practices and policies should be linked to job design. Job design is the analysis, and variation of the content, structure, and environment within which jobs and roles are placed in the social, physical, and organizational context (Morgeson and Humphrey, 2008). So, job design is related to individual, group, and organizational outcomes (Morgeson and Humphrey, 2006) such as job satisfaction. In remote work, and especially in a period of crisis such as the pandemic, job design could be a tool to be implemented with a view to preventing and safeguarding wellbeing. Furthermore, to improve job satisfaction it is necessary to take into account the work of Truxillo et al. (2012) who, by examining the possible joint effects of age and job characteristics, offer guidelines to improve job satisfaction. Our findings also suggest taking into account gender and the presence of minor children. Starting from their literature survey, we propose to act on the following dimensions to increase job satisfaction in working from home: autonomy, task variety and significance, skill variety and specialization, interdependence, social support and feedback. Above all, there must be opportunities to give and receive accurate feedback and support from the organization. The actions and interventions described above can also act on the job demands considered in the study, workload, and social isolation, which most likely were increased during the lockdown, which negatively impacted the quality of life (see hypotheses H1, H6 and H7). For these reasons, as also reported by Bulińska-Stangrecka and Bagieńska (2021), in pandemic times, it could also be useful to create spaces for interaction. The relevance of perceived organizational support, as a job resource, appears to be decisive for discussing the relationship between organization and employees, in the specific case of pandemic management. During the COVID-19 pandemic, many organizations have implemented full-time remote work for their employees in response to the health crisis. Consequently, more extensive remote work support was needed in organizations (e.g., information technology support, timely information, relevant work materials) (Chong et al., 2020; Fernandez and Shaw, 2020) not only to accept such a radical change in working practices but also to cope with working conditions that are not always easy (e.g., overlapping work – family tasks; difficult workload management) (Mäkiniemi et al., 2021). In addition, our findings suggest that individual job-resources (vision about the future, self-efficacy, and commitment to change) have a positive direct (see hypothesis H5) and indirect effect (see hypothesis H3 and H4) on the quality of life. Specifically, self-efficacy and commitment to change have significant indirect effects on quality of life as they are negatively associated with perceived stress (see hypotheses H7b and H7d). Furthermore self-efficacy positively influences job satisfaction (see hypothesis H4b). It can therefore be said that confidence in one’s abilities is not only positively associated with satisfaction (Judge and Larsen, 2001), but more generally leads people to minimize stress (Grau et al., 2001) (see hypothesis H3b). Given our findings, it would appear that employees who are confident in their ability to cope with change are not only better equipped to contribute to the change process, and to manage the stress of change (Cunningham, 2002), but also more satisfied (Judge et al., 1998) (see hypothesis H3d) and this in turn positively affects their evaluation of quality of life (see hypotheses H5c and H7d). Both commitment to change, and vision about the future, also have a significant total effect on the quality of life (see hypotheses H5b, H5c, H7c, H7d, H9c and H9d). As for the commitment to change, it is likely that the positive influence on the perception of quality of life is related to the fact that change is perceived as a process necessary to manage the reorganization of one’s work and lifestyle in general, because of restrictions due to the pandemic. As well as temporal perspective, namely the capacity to project oneself towards the future even in conditions of strong “*presentification*” imposed by the general crisis, is crucial for interpreting the impact of the pandemic upheaval on the work and personal front, and therefore on the perceived quality of life. However, the extreme uncertainty with which the exit from the state of exception was treated in the public management of the pandemic, sometimes with time horizons of a few weeks between one wave and the next, marked by a series of government decrees, has generated a state of perennial uncertainty that has greatly compromised the ability of individuals to plan and project forward, generally triggering the phenomenon of “pandemic fatigue” illustrated by the literature (World Health Organization (WHO), 2020; Patel et al., 2020; Saladino et al., 2020). In fact, once the perception of the state of emergency as temporary and well defined in its contours has ceased, also the request to be able to equally temporary adapt themselves in terms of additional efforts on a personal and work level has missed its point and meaning. So, when the condition of instability has become more and more undefined with respect to the future, it may have given way to anomic drifts and therefore also to a greater perception of stress in relation to one’s own performativity. Therefore, the results of our study confirm that the dynamics of dyscrasia in the comparison between the instantaneous state of emergency and the medium-term goal of a recovery of stability have been amplified by a framework of fragmented, uncertain and variable information during the different phases of pandemic management, with respect to which individual resources of greater or lesser commitment to change may have made a difference. Finally, the existing literature has highlighted the asymmetrical impact of the pandemic emergency on men and women also in the Italian labor market (see e.g., Del Boca et al., 2020, 2021). Our analysis highlights a slightly larger effect for women, with respect to the whole sample, of workload and having young children on perceived stress, and of organizational support, self-efficacy and having children in pre-scholar age on job satisfaction. The gender factor is expressed through a clear process of collision between private care responsibilities and professional functions that has invested most women, drawing on the contents of their “*moral*” careers marked by a priority presence on the domestic front and a complementary one on the working front, thus exasperating them both. In conclusion, although the existing literature has extensively explored the JD-R model and highlighted the effectiveness of personal resources to cope with the efforts required by the job, the psychological and socio-economic consequences generated by the policy response to the pandemic motivate new interest on the topic, especially in the organization-employees relationship. The pandemic has requested organizations to rethink their management logic, pushing them to abandon old managerial models, based on power and control, to embrace a more open and flexible model that focuses not only on innovation and knowledge but also on workers’ wellbeing and quality of life.

## Practical Implications

It seems important to emphasize that the results achieved in this study suggest some practical implications for human resource management. In light of what stressed in the discussion, it would be necessary for organizations to begin to tackle the issue of human resource management more incisively, adopting a more inclusive and differentiated approach to support all employees, and considering the employees’ different needs to better balance personal and work life. In the conditions of uncertainty caused by COVID-19, it is necessary for organizations to anticipate and detect potential risks and problems due to the radical change in both the way of working and the workplace, guaranteeing workers constant and diversified support that allows them to bring ahead of the objectives and the same levels of productivity, but at the same time protect them from the risk of losing wellbeing. In line with the study’s findings of Manuti et al. (2020) it is crucial, within this scenario of important changes in the relationship between employees and organization, to refocus the centrality of the human resource management function within organizations, redefining policies and practices for people management in order to support employees in facing this very difficult moment of uncertainty, further exacerbated by the fear of the prolongation and consequences of the COVID19 pandemic, on health and on the future. As already highlighted by other studies (see for example Feng and Savani, 2020), our results point to some gender difference in the perception of workload and in the detrimental effect of the presence of minors at home on both work stress and job satisfaction. Thus suggesting that working from home could become a new factor influencing gender gaps in work-related outcomes. Therefore, it is increasingly necessary for organizations to consider the possibility of differentiated effects of remote working on different segments of the workforce. It should be noted that, due to the quarantine and isolation measures imposed to contrast the COVID-19 pandemic, the level of anxiety, stress and psychological problems of employees is increasing, thus calling for the development of strategies to improve the physical and mental health of employees as well as mechanisms of communication and support (Gómez et al., 2020). Physical and mental health is the cornerstone of both job satisfaction and effective employee performance (Chanana, 2020; López-Cabarcos et al., 2020; Su et al., 2021), and affects people’s quality of life in general.

## Limitations

The present study has different limitations. In the absence of longitudinal, or even retrospective, data on respondents, we cannot address the issue of individual unobservable heterogeneity that might simultaneously affect the individual propensity to be stressed, unsatisfied with work, and quality of life. Nonetheless, our analysis provides an accurate description of the relationships between JDR controls and quality of life, taking into account the role played by the mediators. In addition, it is foreseeable that other lockdown periods will occur, as on the other hand has happened so far in 2021, therefore it can be expected that working remotely on the one hand will become easier as people get used to the new ways of working. Nonetheless, for some employees, the situation may worsen due to the inability to cope with the new demands of the job. Therefore, a longitudinal study could be useful to better understand perceptual differences of remote working and their impact on quality of life. Because our sample represents only employees working in Italy, this study suffers a lack of generalizability. Future studies may analyse the effect of other job demands and resources, which are specific to different geographic areas and jobs. The present study is based on the JD-R model; future studies may adopt some other framework or may further build on demands and resources mentioned in the present study. The present study is based on self-reported data from employees and hence a future study may endeavor a holistic study by taking perceptions of managers as well.

## Data Availability Statement

The raw data supporting the conclusions of this article will be made available by the authors, without undue reservation.

## Ethics Statement

Ethical review and approval was not required for the study on human participants in accordance with the local legislation and institutional requirements. The patients/participants provided their written informed consent to participate in this study.

## Author Contributions

All authors developed the research project and collected the data. BB, SD, EC, and CC worked on the literature. BB developed the theoretical background. SB and IS performed methods and data analysis. BB, EC, SB, CC, and SD worked on the conclusions and practical implications. BB, SB, and IS reviewed the manuscript. SA reviewed the references.

## Conflict of Interest

The authors declare that the research was conducted in the absence of any commercial or financial relationships that could be construed as a potential conflict of interest.

## Publisher’s Note

All claims expressed in this article are solely those of the authors and do not necessarily represent those of their affiliated organizations, or those of the publisher, the editors and the reviewers. Any product that may be evaluated in this article, or claim that may be made by its manufacturer, is not guaranteed or endorsed by the publisher.
