# Supplementary material for: Don’t Call It Smart: Working From Home During the Pandemic Crisis
Source: Front Psychol. 2021 Sep 30;12:741585. doi: 10.3389/fpsyg.2021.741585 (PMC8515044; doi:10.3389/fpsyg.2021.741585)
Supplement: Supplementary file 1 [file Table_1.docx]

**Table 1**. Estimates from a hybrid model for Quality of Life in a Latent Path Analysis framework.

|  | ***Quality of life*** | | | | | | | | | | |  | ***Job satisfaction*** | | |  | ***Perceived stress*** | | |
| --- | --- | --- | --- | --- | --- | --- | --- | --- | --- | --- | --- | --- | --- | --- | --- | --- | --- | --- | --- |
|  | **direct effect** | | |  | **indirect effect** | | |  | **total effect** | | |  |  |  |  |  |  |  |  |
|  | Coef. | P>z | Std. Coeff. |  | Coef. | P>z | Std. Coeff. |  | Coef. | P>z | Std. Coeff. |  | Coef. | P>z | Std. Coeff. |  | Coef. | P>z | Std. Coeff. |
| *job satisfaction* | 0.072 | 0.002 | 0.14 |  |  |  |  |  | 0.072 | 0.002 | 0.14 |  |  |  |  |  |  |  |  |
| *perceived stress* | -0.665 | 0.000 | -0.39 |  |  |  |  |  | -0.665 | 0.000 | -0.39 |  |  |  |  |  |  |  |  |
| *self-efficacy* | -0.033 | 0.545 | -0.03 |  | 0.101 | 0.000 | 0.09 |  | 0.069 | 0.221 | 0.06 |  | 0.332 | 0.007 | 0.15 |  | -0.117 | 0.000 | -0.18 |
| *vision about future* | 0.323 | 0.000 | 0.36 |  | 0.182 | 0.000 | 0.20 |  | 0.506 | 0.000 | 0.56 |  | 0.453 | 0.000 | 0.26 |  | -0.226 | 0.000 | -0.43 |
| *commitment to change* | 0.066 | 0.351 | 0.04 |  | 0.123 | 0.001 | 0.08 |  | 0.189 | 0.010 | 0.12 |  | 0.351 | 0.063 | 0.12 |  | -0.147 | 0.002 | -0.16 |
| *social isolation* |  |  |  |  | -0.046 | 0.031 | -0.05 |  | -0.046 | 0.031 | -0.05 |  | 0.188 | 0.079 | 0.10 |  | 0.090 | 0.001 | 0.15 |
| *workload* |  |  |  |  | -0.125 | 0.003 | -0.06 |  | -0.125 | 0.003 | -0.06 |  | 0.070 | 0.739 | 0.02 |  | 0.195 | 0.000 | 0.17 |
| *perceived organisational support* |  |  |  |  | 0.045 | 0.074 | 0.05 |  | 0.045 | 0.074 | 0.05 |  | 0.605 | 0.000 | 0.32 |  | -0.003 | 0.926 | 0.00 |
| female | 0.110 | 0.549 | 0.02 |  | -0.097 | 0.260 | -0.02 |  | 0.012 | 0.950 | 0.00 |  | 1.273 | 0.003 | 0.14 |  | 0.283 | 0.008 | 0.11 |
| age | 0.061 | 0.515 | 0.24 |  | 0.002 | 0.964 | 0.01 |  | 0.063 | 0.537 | 0.25 |  | -0.266 | 0.232 | -0.54 |  | -0.031 | 0.573 | -0.22 |
| age squared (/100) | -0.060 | 0.564 | -0.22 |  | -0.003 | 0.942 | -0.01 |  | -0.063 | 0.575 | -0.23 |  | 0.294 | 0.227 | 0.55 |  | 0.037 | 0.550 | 0.23 |
| married | 0.323 | 0.085 | 0.07 |  | -0.029 | 0.722 | -0.01 |  | 0.294 | 0.148 | 0.07 |  | -0.257 | 0.562 | -0.03 |  | 0.015 | 0.889 | 0.01 |
| children (0-6) | -0.249 | 0.287 | -0.05 |  | -0.257 | 0.015 | -0.05 |  | -0.506 | 0.045 | -0.10 |  | -0.747 | 0.174 | -0.07 |  | 0.306 | 0.026 | 0.10 |
| children (6-18) | -0.241 | 0.266 | -0.05 |  | -0.217 | 0.026 | -0.05 |  | -0.457 | 0.051 | -0.10 |  | -0.153 | 0.765 | -0.02 |  | 0.309 | 0.016 | 0.11 |
| children (18 and older) | -0.272 | 0.381 | -0.04 |  | -0.026 | 0.846 | 0.00 |  | -0.299 | 0.378 | -0.04 |  | 0.227 | 0.759 | 0.02 |  | 0.064 | 0.731 | 0.02 |
| univerisity degree | 0.487 | 0.014 | 0.10 |  | -0.054 | 0.537 | -0.01 |  | 0.433 | 0.044 | 0.09 |  | 0.728 | 0.120 | 0.08 |  | 0.160 | 0.173 | 0.06 |
| employed in public sector | -0.068 | 0.697 | -0.02 |  | 0.255 | 0.002 | 0.06 |  | 0.187 | 0.320 | 0.05 |  | 0.318 | 0.438 | 0.04 |  | -0.350 | 0.001 | -0.15 |
|  |  |  |  |  |  |  |  |  |  |  |  |  |  |  |  |  |  |  |  |
| R-squared (for the whole model) |  | 0.739 |  |  |  |  |  |  |  |  |  |  |  |  |  |  |  |  |  |
| R-squared (for each equation) |  |  |  |  |  |  |  |  | 0.569 |  |  |  | 0.370 |  |  |  | 0.553 |  |  |
| N |  | 293 |  |  |  |  |  |  |  |  |  |  |  |  |  |  |  |  |  |
| Log-Likelihood |  | -7020.302 | |  |  |  |  |  |  |  |  |  |  |  |  |  |  |  |  |
| RMSE |  | 0.02 (CI 90%: 0.000-0.093) | | | |  |  |  |  |  |  |  |  |  |  |  |  |  |  |

*Notes*. For estimation we use Stata 16. Standardized coefficients (r) are reported for each variable.
