# Supplementary material for: Don’t Call It Smart: Working From Home During the Pandemic Crisis
Source: Front Psychol. 2021 Sep 30;12:741585. doi: 10.3389/fpsyg.2021.741585 (PMC8515044; doi:10.3389/fpsyg.2021.741585)
Supplement: Supplementary file 2 [file Table_2.docx]

**Table 2**. Estimates from a hybrid model for Quality of Life in a Latent Path Analysis framework (women sub-sample)

|  | ***Quality of life*** | | | | | | | | | | |  | ***Job satisfaction*** | | |  | ***Perceived stress*** | | |
| --- | --- | --- | --- | --- | --- | --- | --- | --- | --- | --- | --- | --- | --- | --- | --- | --- | --- | --- | --- |
|  | **direct effect** | | |  | **indirect effect** | | |  | **total effect** | | |  |  |  |  |  |  |  |  |
|  | Coef. | P>z | Std. Coeff. |  | Coef. | P>z | Std. Coeff. |  | Coef. | P>z | Std. Coeff. |  | Coef. | P>z | Std. Coeff. |  | Coef. | P>z | Std. Coeff. |
| *job satisfaction* | 0.099 | 0.000 | 0.19 |  |  |  |  |  | 0.099 | 0.000 | 0.19 |  |  |  |  |  |  |  |  |
| *perceived stress* | -0.717 | 0.000 | -0.40 |  |  |  |  |  | -0.717 | 0.000 | -0.40 |  |  |  |  |  |  |  |  |
| *self-efficacy* | -0.061 | 0.363 | -0.05 |  | 0.111 | 0.001 | 0.09 |  | 0.050 | 0.481 | 0.04 |  | 0.458 | 0.002 | 0.20 |  | -0.091 | 0.011 | -0.14 |
| *vision about future* | 0.287 | 0.000 | 0.30 |  | 0.222 | 0.000 | 0.24 |  | 0.509 | 0.000 | 0.54 |  | 0.581 | 0.000 | 0.33 |  | -0.228 | 0.000 | -0.44 |
| *commitment to change* | 0.120 | 0.172 | 0.07 |  | 0.112 | 0.019 | 0.07 |  | 0.231 | 0.014 | 0.14 |  | 0.106 | 0.628 | 0.03 |  | -0.141 | 0.009 | -0.15 |
| *social isolation* |  |  |  |  | -0.043 | 0.117 | -0.04 |  | -0.043 | 0.117 | -0.04 |  | 0.286 | 0.016 | 0.14 |  | 0.100 | 0.001 | 0.17 |
| *workload* |  |  |  |  | -0.137 | 0.013 | -0.07 |  | -0.137 | 0.013 | -0.07 |  | 0.204 | 0.395 | 0.05 |  | 0.220 | 0.000 | 0.19 |
| *perceived organisational support* |  |  |  |  | 0.077 | 0.022 | 0.08 |  | 0.077 | 0.022 | 0.08 |  | 0.686 | 0.000 | 0.36 |  | -0.012 | 0.718 | -0.02 |
| age | 0.104 | 0.360 | 0.39 |  | -0.034 | 0.518 | -0.13 |  | 0.070 | 0.572 | 0.26 |  | -0.102 | 0.691 | -0.20 |  | 0.033 | 0.602 | 0.22 |
| age squared (/100) | -0.105 | 0.405 | -0.36 |  | 0.036 | 0.530 | 0.12 |  | -0.069 | 0.618 | -0.23 |  | 0.112 | 0.690 | 0.20 |  | -0.035 | 0.618 | -0.21 |
| married | 0.209 | 0.337 | 0.05 |  | -0.127 | 0.207 | -0.03 |  | 0.082 | 0.731 | 0.02 |  | -0.502 | 0.302 | -0.06 |  | 0.107 | 0.371 | 0.04 |
| children (0-6) | -0.124 | 0.674 | -0.02 |  | -0.419 | 0.004 | -0.07 |  | -0.543 | 0.087 | -0.10 |  | -1.180 | 0.069 | -0.11 |  | 0.421 | 0.008 | 0.13 |
| children (6-18) | -0.279 | 0.283 | -0.06 |  | -0.281 | 0.024 | -0.06 |  | -0.560 | 0.047 | -0.11 |  | -0.260 | 0.653 | -0.03 |  | 0.356 | 0.012 | 0.13 |
| children (18 and older) | -0.235 | 0.515 | -0.03 |  | -0.027 | 0.870 | 0.00 |  | -0.262 | 0.509 | -0.04 |  | 0.163 | 0.841 | 0.01 |  | 0.060 | 0.764 | 0.01 |
| univerisity degree | 0.502 | 0.036 | 0.10 |  | -0.013 | 0.907 | 0.00 |  | 0.489 | 0.062 | 0.09 |  | 0.612 | 0.257 | 0.06 |  | 0.103 | 0.440 | 0.04 |
| employed in public sector | -0.115 | 0.597 | -0.03 |  | 0.380 | 0.001 | 0.09 |  | 0.265 | 0.258 | 0.06 |  | 0.504 | 0.295 | 0.06 |  | -0.460 | 0.000 | -0.19 |
|  |  |  |  |  |  |  |  |  |  |  |  |  |  |  |  |  |  |  |  |
| R-squared (for the whole model) |  | 0.760 |  |  |  |  |  |  |  |  |  |  |  |  |  |  |  |  |  |
| R-squared (for each equation) |  |  |  |  |  |  |  |  | 0.585 |  |  |  | 0.415 |  |  |  | 0.589 |  |  |
| N |  | 216 |  |  |  |  |  |  |  |  |  |  |  |  |  |  |  |  |  |
| Log-Likelihood |  | -5024.306 | |  |  |  |  |  |  |  |  |  |  |  |  |  |  |  |  |
| RMSE |  | 0.048 (CI 90%: 0.000-0.122) | | | |  |  |  |  |  |  |  |  |  |  |  |  |  |  |

*Notes*. For estimation we use Stata 16. Standardized coefficients (r) are reported for each variable.
